# Ion Channels and Neurological Disease

**DOI:** 10.3390/life14060758

**Published:** 2024-06-13

**Authors:** Carlo Musio

**Affiliations:** Institute of Biophysics—IBF, National Research Council—CNR, Via Sommarive 18, 38123 Trento, Italy; carlo.musio@cnr.it

## 1. Introduction

Ion channels are key elements in the control of membrane physiology and neurotransmission because ionic fluxes assure neuronal signal propagation across and between neurons through synaptic transmission [1,2]. The pathophysiology of ion channels may originate from either mutations of gene encoding components of the channel structure (channelopathy) or secondary dysfunctions; both conditions affect the intrinsic excitability of the cell and synaptic functions, leading to the pathophysiological signs of disease [3]. Accordingly, ion channels are considered suitable drug targets within modern pharmacology [4].

Neurological disorders represent pathological conditions that directly affect the nervous system or brain functions that produce single or composed neurodevelopmental, motor, sensory, or cognitive organic impairments whose etiology can be genotypical or idiopathic [5]. Most currently known neurological diseases, mainly neurodegenerative diseases (NDDs), exhibit alteration of neuronal excitability due to the dysfunction of the molecular and/or functional features of ion channels. In the majority of NDDs, the pathogenic role of ion channels has been widely demonstrated either for channelopathies or secondary dysfunction [6]. Nevertheless, the link between ion channel alterations that induce neuronal excitability and the onset of disease has been neglected in some disorders, while for others, this area of study is growing rapidly.

The aim of this Special Issue is to provide updates on the state of the art and new achievements in research on altered structure–function relationships in the ion channels that affect the pathophysiology of neurological disease. We are particularly interested in methods of drug screening and targeting that will allow for the development of novel therapeutic avenues for treating and alleviating these mostly incurable diseases. Multi- and inter-disciplinary research contributions—often combining structural, functional, and/or pharmacological approaches with different methods/techniques—are presented.

## 2. The Special Issue

This Special Issue, entitled “Ion Channels and Neurological Disease” [7], belongs to *Life*’s “Pharmaceutical Science” section [8] and is a collection of ten peer-reviewed articles (seven reviews and three original articles) covering dysfunctions of the main typologies (sodium, potassium, calcium, chloride) of neuronal ion channels. Furthermore, new data on GABA receptors, nucleoporin, and the ion channels of neuroglia are reported. The main related neurological disorders include Alzheimer’s disease, glioblastoma, Parkinson’s disease, cerebellar ataxias. The Special Issue’s contributions are grouped below ordered by functional classification, while they are listed at the end of the editorial along with the web site order.

### 2.1. Sodium (Na^+^) Channels

A review on the impairments of voltage-gated sodium channels in neurological disorders opens the issue. Starting from the channels’ conserved genes, *SCN1A*, etc. to the encoded proteins Na_v_1.1, etc., a comprehensive survey outlines the structure, the function and the role of the Na_v_ channel mutations, emphasizing pharmacological therapeutic approaches in several disorders, like migraine, autism, and Alzheimer’s (Contribution 9).

Accordingly, a review on the same channel types focuses their role in the pathophysiology of Alzheimer’s disease. In particular, their role in mediating and tuning functional and altered neuronal excitability and the attenuation of hippocampal hyperactivity (which seems to ameliorate cognitive deficits) are considered promising elements to be addressed by potential therapeutic interventions (Contribution 6).

### 2.2. Potassium (K^+^) Channels

Contribution 7 synthesizes all the recent literature from the Shakkottai lab, emphasizing the role of ion channels (and potassium ones in particular) in the dysregulation of neuronal excitability in several spinocerebellar ataxias (SCAs), a specific subset of polyglutaminic neurodegenerative diseases. Therefore, through the targeting of those channels, treatments that restore the altered intrinsic membrane excitability of cerebellar Purkinje neurons emerge as an important pharmacological and neurotherapeutic avenue.

### 2.3. Calcium (Ca^2+^) Channels

Another original article and a review address the role that dysregulation of neuronal calcium channels plays in the pathogenesis of Alzheimer’s disease (AD).

Said original research article (Contribution 1) reports the functional characterization of calcium and glutamate phenotypes involved in the pathogenesis of *PSEN1*-mutated familial Alzheimer’s disease (FAD). They measured Ca^2+^ response dynamics in induced pluripotent stem cell (iPSC)-derived neurons carrying *PSEN1* mutations to glutamate, NMDA, AMPA and kainate, showing that alterations in Ca^2+^ and glutamate signaling can be considered an early functional FAD phenotype.

The review (Contribution 10) discusses a new challenging pathogenic element of AD which could reside in the functional feedback between Ca2+ signaling and lysosomal/autophagic dysfunctionality. Targeting and fine-tuning this functional link with drugs could represent a novel route to countering AD and (possibly) NDDs.

### 2.4. Chloride (Cl^−^) Channels

Contribution 8 outlines the biophysical properties and the functional role of five types of transporters belonging to the voltage-gated ChLoride channels (CLC) family and located in the membranes of endosomes and lysosomes, which are intracellular organelles regulating the homeostatic and autophagic processes of the cell. These channels are crucial for anion/proton exchange and pH regulation: their mutations have been identified within the pathogenesis of several diseases, including neurodegenerative and neurodevelopmental disorders.

### 2.5. GABA Receptors and Currents

Two more contributions deal with the activation of GABA receptors and the biophysical properties of GABA currents.

The former (Contribution 3) group carried out an intriguing form of neural tissue preparation (i.e., slices of turtle spinal cord), exploring the activation of GABA_B_ receptors in the terminals and axons of dorsolateral funiculus (DLF) eliciting functionally antagonist post-synaptic potential in motor neurons. The paper reports indications that GABA_B_ receptors are activated by environmental GABA, the concentration of which is regulated by their release from interneurons and astrocytes.

The latter (Contribution 4) focuses on the biophysical alterations in GABA_A_ reversal potential, showing them to be crucial features in several perturbed conditions, as a consequence of disequilibrium in the NKCC1-KCC2 (chloride–cation transporters) activity ratio. As GABA_A_ current alteration has been identified in several neurological diseases, the paper reports imbalance in chloride–cation transporters to be a therapeutic target.

### 2.6. Neuroglia Channels and Channel-Modulating Nucleoporins

Two articles conclude this roundup on ion channels and neurological disease.

Contribution 5 provides a very thorough and up-to-date review of the functional roles of ion channels and ionotropic receptors in functional and pathological astrocytes, focusing mainly Alzheimer’s disease (AD) and glioblastoma brain tumor (GBM). The authors underline that better collective understanding of ion channels and ionotropic receptors in astrocytes, which are involved in the above diseases, is required to develop novel therapeutic interventions and new strategies for treating brain disorders.

In Contribution 2, the tuning of neuronal excitability by nucleoporin NUP358 in mouse primary cortical neurons is reported; this regulating activity is exerted via a voltage-gated sodium channel. The authors stress the role of altered neuronal excitability as a potential key player in the pathogenesis of neurological disorders and present a window into ion channel pathophysiology in neurodegenerative diseases.

## 3. Conclusions

This Special Issue, “Ion Channels and Neurological Disease”, presents several intriguing and up-to-date aspects of ion channels’ role in the pathogenesis—and/or the pathological phenotype—of neurological disorders. Nowadays, there is a general consensus on the role exerted by ion channels in determining alterations in neuronal excitability. Thus, the altered structure/function relationship of ion channels can be considered a common feature of several neurological disorders; we may use this common feature to develop new therapeutic tools and avenues. 

Finally, this Special Issue has received good attention and visibility in terms of total and single article views. Accordingly, a second edition of the Special Issue and a printed book of the first edition are in preparation. The second edition, co-edited by Carlo Musio (IBF-CNR Trento, Italy) and Marzia Martina (NRC Ottawa, Canada), is already open and in progress; further information and details are available on the official website [9]. 
